# Innovative molecular networking analysis of steroids and characterisation of the urinary steroidome

**DOI:** 10.1038/s41597-024-03599-0

**Published:** 2024-07-24

**Authors:** Ting Chen, Justine Massias, Samuel Bertrand, Yann Guitton, Bruno Le Bizec, Gaud Dervilly

**Affiliations:** 1 Oniris, INRAE, LABERCA, Nantes, 44307 France; 2 Nantes Université, Institut des Substances et Organismes de la Mer, ISOMER, UR 2160, F-44000 Nantes, France; 3 Nantes Université, École Centrale Nantes, CNRS, LS2N, UMR 6004, F-44000 Nantes, France

**Keywords:** Agriculture, Biomarkers

## Abstract

Steroids are cholesterol-derived biomolecules that play an essential role in biological processes. These substances used as growth promoters in animals are strictly regulated worldwide. Targeted assays are the conventional methods of monitoring steroid abuse, with limitations: only detect known metabolites. Metabolism leads to many potential compounds (isomers), which complicates the analysis. Thus, to overcome these limitations, non-targeted analysis offers new opportunities for a deeper understanding of metabolites related to steroid metabolism. Molecular networking (MN) appears to be an innovative strategy combining high-resolution mass spectrometry and specific data processing to study metabolic pathways. In the present study, two databases and networks of steroids were constructed to lay the foundations for the implementation of the GNPS-MN approach. Steroids of the same family were grouped together, nandrolone and testosterone were linked to other analogues. This network and associated database were then applied to a few urine samples in order to demonstrate the annotation capacity in steroidome study. The results show that MN strategy could be used to study steroid metabolism and highlight biomarkers.

## Background & Summary

**Fig. 1:**
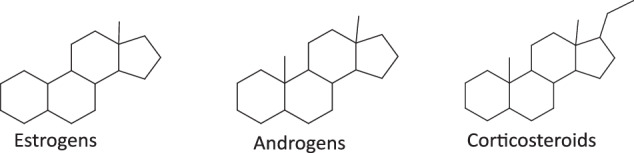
The chemical skeleton of estrogens, androgens and corticosteroids.

Steroid hormones are non-polar compounds with a perhydro-1, 2-cyclopentanophenanthrene ring system. Based on their specific chemical structures, they can be divided into androgens (C19), estrogens (C18), glucocorticoids (C21), and mineralocorticoids (C21) (Fig. 1). Endogenous steroids, *e.g*. testosterone, are naturally produced by the organism and play a significant role in physiological activities, such as physical development, metabolic homeostasis, and sexual maturation. For specific applications (*e.g*. medical, veterinary, etc.), certain steroids are the subject of chemical synthesis, either the same structures as those naturally produced or strictly exogenous variants^1–4^. In particular, endogenous and exogenous steroids have been used as anabolic agents with benefits on growth in animal husbandry over the past 60 years. The steroids of interest in breeding are mainly androgens (*e.g*. testosterone, nandrolone, boldenone) or estrogens such as estradiol. The presence of residues of these substances in foodstuffs from treated animals raises health questions for the consumer since steroids are endocrine disruptors^5–9^. For these reasons and because of the risk assessment, notably carried out at the global level by the Joint FAO/WHO Expert Committee on Food Additives (JECFA), measures have been taken in various regions of the world going as far as banning its use in Europe in particular. JECFA has defined an Acceptable Daily Intake (ADI) of 0–2 µg/kg bw/d and 0–0.05 µg/kg bw/d for testosterone and estradiol (52nd JECFA, 1999), respectively, as well as Maximum Residue Limits (MRL) in certain animal tissues. To comply with these provisions, control measures, in particular based on analytical strategies, are put in place by analytical laboratories to monitor the presence of steroids or their metabolites in animal products^10,11^.

Conventional analytical strategies to monitor steroids are targeted analyses relying on Gas Chromatography Mass Spectrometry (GC-MS) or Liquid Chromatography Mass Spectrometry (LC-MS). The targeted methods can monitor the parent compounds or their direct metabolites, *e.g*. phase I or phase II metabolized compounds^5,7,12–18^. If these strategies are very effective, the fact remains that they come up against certain limits, such as the administration of unknown synthetic steroids or the use of low-dose steroid cocktails, these two doping strategies put at risk the selectivity and sensitivity properties of current approaches. It is in this precise context and to guarantee a high level of food safety that the search for new strategies to optimize control performance takes place^19,20^. It is known that the administration of steroids leads to an overall modification of the urinary steroidome, which is composed of hundreds of steroidal compounds whose profiles (qualitative and quantitative) are inter-correlated. This is why it may be interesting to explore this specific chemical space in a more comprehensive way in order to reveal new biomarker candidates to detect a greater number of compounds and their effects over a wider detection window^21,22^. As steroids represent a large family of compounds and their metabolites are just as numerous, the administration of one steroid has consequences on the profile of others. The main challenge is still to improve the detection performance further to find more new metabolites related to steroid metabolism. Hence, the global strategy to study the relationships between lots of steroids from different families is very interesting.

**Fig. 2:**
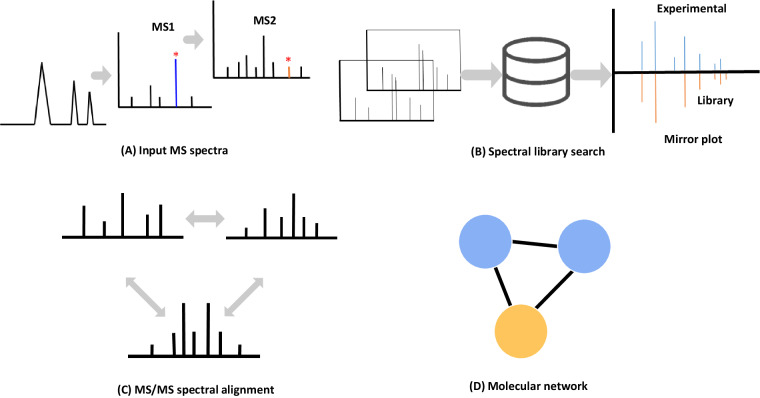
The workflow of the MN. (**A**) The most intense ions detected in MS1 are selected and fragmented to acquire MS/MS spectral data. (**B**) To find a good spectral match, each MS/MS spectrum from the dataset is searched against spectral libraries. (**C,D**) Spectra with high similarities are grouped and form molecular networks based on the spectral similarity (cosine score).

Innovative approaches based on tandem mass spectrometry data were recently developed to point out structural similarity among detected ions, such methodology is Molecular Networking (MN), which has become a popular tool in the analysis of Tandem Mass Spectrometry (MS/MS)-based metabolomics studies^23–26^. As MN organizes chemically similar compounds into clusters and reveals the relationship between molecules, such as a shared sub-structure between metabolites, it can facilitate the recognition of patterns at a chemical family level and also enhance the structural characterization of multiple connected metabolites. The workflow of MN is shown in Fig. 2. Global Natural Product Social Molecular Networking (GNPS, https://gnps.ucsd.edu), which is a platform that serves both as data analysis with MN and data repository as well as library capabilities, is currently the only online infrastructure that provides MN^25,27–30^. Recently, GNPS-MN has become an essential toolbox for metabolomics-type studies. Indeed, it compares pairs of MS/MS spectra based on their similarities and connects them to MS/MS reference spectral libraries. Then, MN allows further propagation of annotations via mass spectral relationships. Visualization of molecular networks in GNPS represents each spectrum as a node, with spectrum-to-spectrum alignments as edges between nodes. The clusters generated in the molecular networks could then bring together metabolites belonging to the same chemical families or sharing closely related chemical structures, which could apply to steroids and thus help to study new steroid metabolites. GNPS-MN was first introduced to analyse LC-MS data using a method referred to as classical molecular network. Then, Feature-Based Molecular Networking (FBMN) was developed to improve the classical approach by incorporating MS1 information, which can distinguish isomers that might have remained hidden and facilitate spectral annotation^29,31^. Subsequently, GNPS-MN was introduced into GC-MS data, with the workflow including GC-MS deconvolution, alignment and mass spectral library matching^27^.

The strategy of MN thus appears to be an interesting approach for globally studying compounds such as steroids, whose chemical structures are close since they are derived from a parent hydrocarbon structure derived from cholesterol^32^. Steroids are thus expected to provide similar fragmentation patterns that may be used to create networks using a MN approach. Currently, to our knowledge, no research has been carried out on urinary steroid profiles based on MN strategy. Meanwhile, MN carried out on GNPS has shown good application prospects in metabolomics studies. The aim of this study was, therefore to explore the MN-based strategy to enable further exploration of steroid metabolism. The first step in such a study is to construct a large (n > 120 steroids) database forming a steroidal network before applying it to samples of interest.

## Methods

### Reference substances and preparation

All reference steroids (n = 88) were acquired from steraloids (Newport, RI, USA), including *epi*-19-nortestosterone, 19-nortestosterone, 19-norandrosterone, 19-noretiocholanolone, 19-nortestosterone sulfate, estradiol, testosterone, androstenedione, 5β-estran-3α, 17α-diol, 5α-estran-3β, 17α-diol, 5α-estran-3α, 17β-diol, cortisone, dexamethasone, prednisolone, etc. Each steroid stock solution was prepared at 100 µg/mL in ethanol. The working standard solutions were prepared by the dilution of stock standard solutions in methanol. The preparation of the reference compounds for LC-HRMS involved analysis of 88 steroid reference compounds. The working solution was 10 µg/mL, and the injection volume was 5 µL. For GC-HRMS analysis, 27 steroid reference compounds have been prepared in a working solution at 10 µg/mL. These 27 steroids were then derivatized 40 min at 60°C with 20 µL MSTFA-TMIS-DTE (1000:5:5, v/v/w). Afterwards, the compounds were ready for injection. All of the solutions were stored in amber glass vials at −20 °C.

### Urine samples and preparation

The urine samples were collected from bovine treated with steroids and already stored at the biobank of the laboratory at −20 °C. Bovine urine samples were prepared as follows and according to previously publications^14,33^. Briefly, 10 mL of urine samples were added with 10 ng 17β nandrolone-d_3_ and 17β-estradiol-d_3_, 1 mL acetate buffer (2 M, pH 5.2) and 200 µL β-glucuronidase from purified Helix pomatia (Sigma-Aldrich, St. Quentin Fallavier, France). Hydrolysis was performed over 15 h at 52 °C. Urine samples were centrifuged (10 min, 1000×g) before purification on SPE Envi-ChromP. Then, cartridges were conditioned with 6 mL ethyl acetate, 6 mL methanol and 6 mL water. The extraction was applied to the column. The phase was washed with 3 mL water and then 2 mL hexane. The high vacuum was applied before and after each washing. Steroids were eluted with 14 mL hexane/diethyl ether (70:30, v/v), which were evaporated to dryness under a gentle stream of nitrogen. After hydrolysis, 0.5 mL of 1 M of NaOH was added. Liquid/liquid extractions in an alkaline medium phase performed with 4 mL hexane/diethyl ether (70:30, v/v) permitted the extraction. The extraction was centrifuged for 1 min, 700 g. The residue supernatant was applied onto an SPE silica column conditioned with 6 mL hexane. The column was washed with 3 mL hexane/ethyl acetate (75:25, v/v) and then 8 mL hexane/ethyl acetate (85:15, v/v). The analytes were eluted with 20 mL hexane/ethyl acetate (60:40, v/v), which were evaporated to dryness under a gentle stream of nitrogen. After that, 10 ng norgestrel (Sigma-Aldrich) was added as an external standard. Finally, the urine samples were derivatized for 40 min at 60 °C with 20 µL MSTFA-TMIS-DTE (1000:5:5, v/v/w). Two µL samples were injected into the GC-Q- Exactive.

### Chemicals and reagents

All reagents and solvents used in this study were of analytical grade unless otherwise specified. Acetonitrile was purchased from Honeywell Chromasolv (Bucharest, Romania). Formic acid (eluent additive for LC-MS) was acquired from LGC Standards GmbH (Wesel, Germany). Isotope-labeled internal standards, namely, L-lecine-5,5,5-d_3_, L-tryptophan-2,3,3-d_3_, indole-2,4,5,6,7-3-acetic acid, and 1,14-tetradecanedioic-d_24_ acid were purchased from sigma-Aldrich (Saint Quentin Fallavier, France). MSCAL6 Proteo Mass LTQ/FT-Hybrid, standard mixtures used for calibration of the MS instrument (positive, negative ionization mode) were obtained from Sigma-Aldrich (Saint Fallavier, France). Ultra-pure water was acquired from VWR (Fontenay-sous-Bois, France). Envi-ChromP and silica (0.5 g and 1 g stationary phase, respectively) solid-phase extraction (SPE) cartridges were from Carlo Erba Réactifs SDS (Val de Rueil, France). Derivatisation reagents N-methyl-N-(trimethylsilyl)-trifluoroacetamide (MSTFA), dithio-threitol (DTE) and trimethyliodosilane (TMIS) were purchased from Acros Organics (Geel, Belgium). The internal standards used were 17β-nandrolone-d_3_ (17β-NT-d_3_), 17β-estradiol-d_3_ (17β-E2-d_3_) from NARL reference materials (Pymble, Australia).

### Data acquisition

88 steroid reference compounds were characterized on LC-Q–Exactive and the analysis was performed according to previously reported LC-based methods for steroids analysis^20,33,34^. The chromatographic separation was performed on an Acquity UPLC® System from Waters®, C18 column (Acquity UPLC® BEH C18, 2.1 × 100 mm, 1.7 mm; Waters®). Separations were carried out at 50 °C under gradient elution conditions. Elution solvents were 0.1% formic acid in water (A) and 0.1% formic acid in acetonitrile (B). The mobile phase was supplied at a flow rate of 0.6 mL/min. Two gradient programs were used for analysing different kinds of steroids: one gradient for characterizing steroid esters and progestogens standards, while another gradient program was for characterizing androgens as well as estrones and others. The elution gradient (A/B, *v/v*) of androgens, estrones characterization was as follows: 95:5 between 0 and 0.3 min, 57:46 at 9.6 min, 0:100 from 13.5 to 15.5 min, and 95:5 from 16 to 19.5 min. At the same time, the elution gradient (A/B, *v/v*) for characterizing steroid esters and progestogens standards was as follows: 50:50 from 0 to 2 min; 90:10 at 9.6 min, 0:100 from 13.5 to 15.5 min, 50:50 from 16 to 19.5 min. The acquisition was performed on an Exactive–Exactive system (Thermo Fisher Scientific, Bremen, Germany) in positive and negative electrospray ionization mode (ESI + /–). The spectrometric parameters are as follows: spray voltage 3.0 kV, capillary temperature 350 °C, sheath gas flow rate 55 U, gas flow rate 10 AU, sheath gas flow rate 55 AU, and the column oven temperature 50 °C. The MS settings were described as follows: The full scan mass spectra were acquired from 66.7 to 1000 *m/z* with a mass resolution of 70 000 in centroid mode, maximum inject time 200 ms, 1e6 AGC target and the 0 to 19.5 min runtime. The integrated Xcalibur software version 4.1 was used for data acquisition and analysis. Background ions can be evaluated and put onto an “exclusion list” to increase the likelihood of obtaining MS2 spectra. Additionally, a separate injection acquired by data-dependent MS2 (DDMS2) for a set of precursor ions (exclusion lists) in the positive or negative was performed to collect more diagnostic MS2 spectra. The parameters of DDMS2 were as follows: 17 500 resolution, 1e5 AGC target, maximum inject time 60 ms, loop count 5, Isolation window 1.0 m/z, collision energy 10, 30, 60; dd setting (Minimum AGC target 6.00e3, Intensity threshold 1.0e5, Charge exclusion 3–8, >8, dynamic exclusion 3.0 s).

Twenty-seven steroid reference compounds were also characterized using a GC-EI-Q–Exactive, according to analytical parameters already reported^15,35–37^. In this sense, 1 µL of derivatized steroid standards were injected into the GC injector at 250 °C by the robotic arm TriPlu^TM^ RSH autosampler (Thermo Scientific^TM^, Bremen, Germany). A flow rate of 5 mL/min of helium in split flow (20.0 mL/min) was applied. The chromatographic separation was performed on a TRACE^TM^ 1310 gas chromatography instrument (Thermo Scientific^TM^, Austin, TX, USA). Helium carrier gas at a flow rate of 1 mL/min was applied for the separation on OPTIMA 5-MS Accent column (30 m length 0.2×5 mm i.d. 0.25 μm, SN 1205365). The temperature gradient program was set as follows: the temperature was initially held at 120 °C for 2 min, a 15 °C/min ramp rate was applied up to 320 °C, and the final temperature of 320 °C was held for 13 min, and the total run time was 20 min. Eluting peaks were transferred through an auxiliary transfer temperature of 320 °C into a Q Exactive^TM^-GC mass spectrometer (Thermo Scientific^TM^, Bremen, Germany). Electron ionization (EI) at 70 eV energy and emission current of 50 µA was used as ionization mode, setting an ion source temperature of 300 °C. Data was acquired in full scan mode and PRM mode with a mass range of *m/z* 66.7–1000 and a resolving power of 120 000 at *m/z* 200. AGC Target was set at 5 × 10^5^ ions with an automatic filling limit and maximum IT at 500 ms. The instrument was controlled by Xcalibur software version 4.1.

The urine samples were analysed under the same conditions as those applied for the characterisation of steroid reference compounds on the GC-HRMS platform.

### Database constitution

**Fig. 3:**
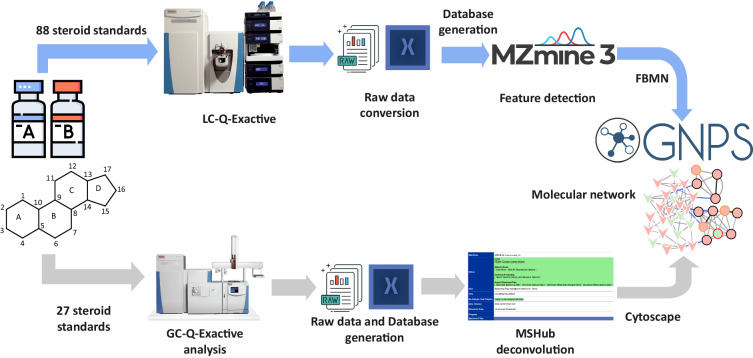
The workflow of database constitution and molecular network.

A database of 88 steroid reference compounds acquired on LC-Q-Exactive was generated, including molecular formula, exact mass, retention time and detected ion in positive and negative modes. Xcalibur version 4.3 software was used to integrate chromatographic peaks acquired in full scan mode with a mass tolerance of 5 ppm. The workflow has been developed and relies on a combination of open bioinformatics tools, including MZmine (https://github.com/mzmi ne/mzmine2/releases)^38–40^, Cytoscape (Version 3.8, https://cytoscape.org/)^41^ and GNPS Web-platform^28^, the workflow of database and molecular networking constitution is shown in Fig. 3. Statistical analysis is done predominantly from MS1-based peaks with a specific accurate mass-to-charge (*m/z*) ratio, described as features of MS-based metabolomics studies. Feature-based molecular network (FBMN) can link MS1 intensities derived from LC-MS features with MS2 information from molecular networking, which bridges the gap between MS1 abundance and MS2 qualitative information compared to classical molecular networking^42^. So FBMN job was performed on the GNPS platform (https://gnps.ucsd.edu). The database constitution procedure of 88 steroid standards, which were acquired on LC-Q–Exactive is as follows. Firstly, the 88 standards files in. raw were converted to centroided.mzML format using MSconvert (version 3.0.20248, http://proteowizard.sourceforge.net/downloads.shtml)^43^. Secondly, a feature detection and alignment tool, MZmine (version 2.53) was used to process the data, which allowed the annotation of steroid isomers^40^. The step allowed the detection of the spectral features across the chromatographic fractions, so *Targeted Feature Detection* was used to detect all the features. The parameters are as following: mass detector = Centroid, intensity tolerance = 50, *m/z* tolerance = 0.05 & 10.0 ppm, retention time tolerance = 2.0 min, algorithm = Wavelets (ADAP), *m/z* centre calculation = MEDIAN, *m/z* range for MS2 scan pairing = 0.1 Da, RT range for MS2 scan pairing = 0.2 min, *S/N* threshold = 10, *S/N* estimator = intensity window SN, feature height = 1 min, area threshold = 15, peak duration range = 0.02–1.0 min, RT wavelet range = 0.01–0.02, *m/z* tolerance = 0.001 & 5.0 ppm, retention time tolerance = 0.05 min. Then two files were exported: *a feature quantification table* (.CSV format) and a MS2 spectral summary (.MGF format). The feature quantification table contains information about features of 88 steroid standards including retention time, intensity, *m/z* value and feature ID (unique identifier) for each feature. The MS2 spectral summary contains a list of MS2 spectra, with one representative MS2 spectrum per feature. Thirdly, the data was achieved by analysing MS/MS data on the GNPS Web platform^28^. The parameters of FBMN are as follows: precursor ion mass tolerance = 0.02 Da, fragment ion mass tolerance = 0.02 Da, minimum cosine score = 0.5, and maximum analogue search mass difference = 100. The analogue search mode was used by searching against MS/MS spectra with a maximum difference of 100 in the precursor ion value. The resulting network was filtered based on edges, and the edges between two nodes were retained in the network only if each node appeared in the respective top 10 most similar nodes of the other node. The spectra in the network were compared against GNPS spectral libraries and our library built in this study. The molecular network job is available on the GNPS platform: https://gnps.ucsd.edu/ProteoSAFe/status.jsp?task = 1b18a9f9c1e649e59deb186ffa020c49. Finaly visualization and analysis of GNPS-generate molecular networks can be performed in Cytoscape (Version 3.8), open-source software for visualizing complex networks^41^. All the dereplicated steroid standards were assigned within the network based on LC-Q-Exactive, MS2 and retention time matching. Adduct connection was manually introduced in the network to provide an ion identity molecular network^44^.

A database was also generated for data acquired on GC-Q-Exactive including retention time, MS, and MS2 information extracted manually by using Xcalibur version 4.3. The 27 standards files in. raw were also converted to centroided.mzML format using MSconvert (version 3.0.20248). FTP client (WinSCP for Windows) was used to upload data files to MassIVE, a public repository. A repository scale analysis infrastructure for GC-MS data enables the creation of networks within the GNPS molecular networking platform. The community infrastructure can be accessed at https://gnps.ucsd.edu, under the heard “GC-MS EI Data Analysis”. MS Hub algorithms (https://gnps.ucsd.edu/ProteoSAFe/static/gnps-splash.jsp) were used for data processing deconvolution. Then, the.mgf file was generated by GNPS-MSHub, including the deconvoluted spectra with aligned retention time and feature table of the peak of the feature across all files, and then searched against the public libraries and private libraries generated in this study. The MSHUB-GC job and molecular network job are available on the GNPS platform: https://gnps.ucsd.edu/ProteoSAFe/status.jsp?task=1c23aec9a10848d3ae24c8554fd81071#; https://gnps.ucsd.edu/ProteoSAFe/status.jsp?task=6ec04b438b444203b00edc096fa11015. The molecular network was also visualized in the Cytoscape (Version 3.8, https://cytoscape.org/)^41^.

### Molecular network construction based LC-HRMS database

**Fig. 4:**
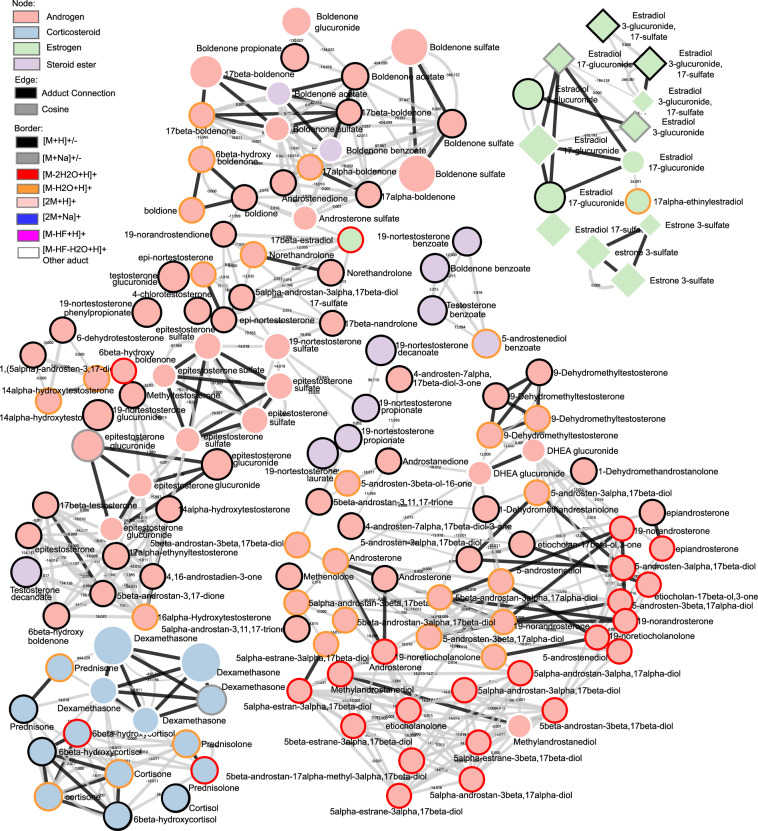
The visualization of the global LC-HRMS steroid network involving 88 steroid reference compounds. The cosine score is 0.5. The node size was marked according to m/z; steroid families were marked in different colors; the edges were also marked in different colors, which represent the similarities of MS/MS; and the grey edge represents cosine between two nodes; black edge represents the adduct connection; node borders were marked in different colors according to adducts. The ellipse represents the node in ESI “+”, while the diamond represents the node in “–”. The size of the node represents the precursor mass.

The database of LC-HRMS involves formulae, accurate mass, retention time, MS1 and MS2 spectra of 88 reference steroid compounds, which correspond to 198 spectra due to multiple detected adducts-producing spectra. These steroids could be separated from 4.6 to 14.56 min on LC-Q-Exactive Most of the steroids could be detected in positive mode, especially [M + H]^+^, [M–2H_2_O + H]^+^ and [M–H_2_O + H]^+^, such as 19-nortestosterone, androsterone, estradiol and boldenone. The group of testosterone steroids can be only detected in [M + H]^+^, *e.g*., *epi*-testosterone, testosterone benzoate, testosterone decanoate and 4-chlorotestosterone. As for some androgens, the known metabolites of 17β-nandrolone could be detected in [M–H_2_O + H]^+^ and [M–2H_2_O + H]^+^, including estranediols, 19-norandrostendione and 19-norandrosterone. Six steroids could be detected in both positive mode and negative mode, 19-nortestosterone sulfate, 19-nortestosterone glucuronide, estradiol 3-glucuronide, 17-sulfate, estradiol 17-sulfate, estradiol 3-glucuronide, estradiol 17-glucuronide. Furthermore, one interesting point is that estradiol 3-glucuronide, 17-sulfate and estradiol 17-glucuronide can be only detected in negative mode, [M–2H]^2–^, [M–H]^–^, and [M–Na]^–^, [2M-Na]^–^, respectively. Thus, the database was used to construct the molecular network. Figure 4 describes the molecular network involving 88 reference steroids presented in circular-shaped nodes. The molecular network was constructed based on the FBMN job, which was performed on GNPS. This molecular network was built on the fundamental observation that structurally related molecules share fragment ion patterns when subjected to MS2 fragmentation methods. Structurally related molecules yield comparable MS2 spectra due to the commonalities in their structures and are represented by separate nodes that connect within the network via edges. In the molecular network, each cluster can be found with the same numbering in the.mgf file, and each node has its spectra information and identification. The compound annotation of this molecular network is based on the steroid standard database generated in this study.

**Fig. 5:**
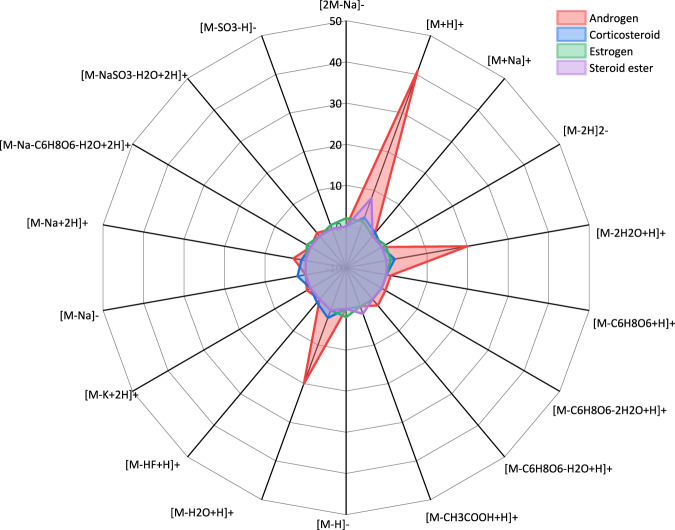
Comparison of chemical species (adducts) detected according to the different steroid families studied.

**Fig. 6:**
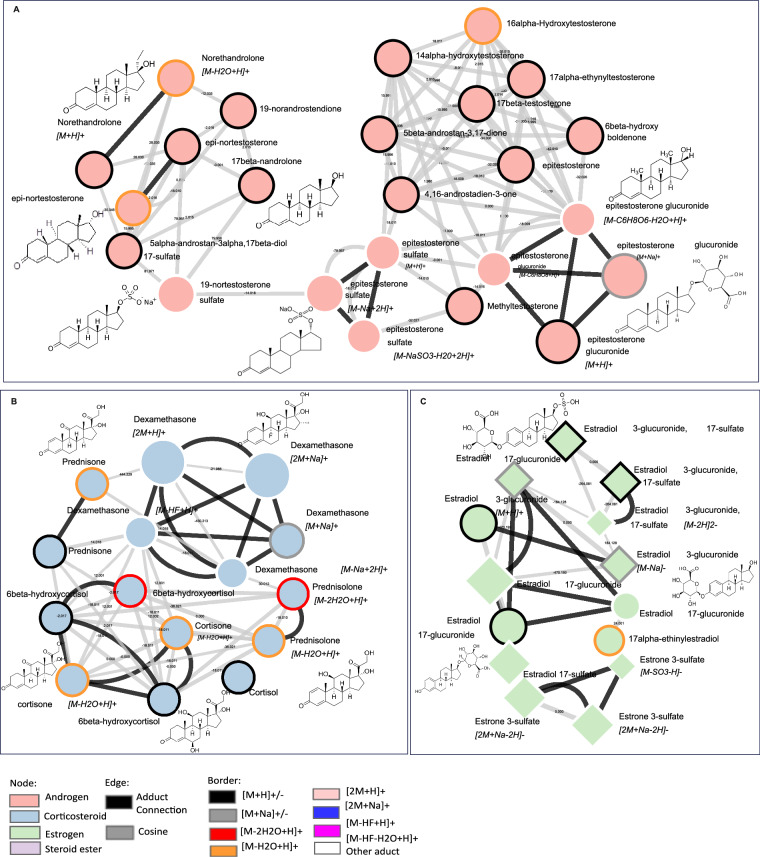
Zooming in on parts of the network obtained with LC-HRMS data and involving structurally related steroids show similar fragmentation patterns (**A**–**C**). The edges were also marked in different colors, which represent the similarities of MS/MS; and the grey edge represents cosine between two nodes; black edge represents the adduct connection; node borders were marked in different colors according to adducts. The ellipse represents the node in ESI “+”, while the diamond represents the node in “−”. The size of node represents the precursor mass.

Because MN allows for the identification of molecular families corresponding to clusters in the network, the spectral similarity is calculated through the cosine score. In this study, the steroid compounds can be observed depending on the relationship between structures in the global molecular network. Overall, the obtained molecular network tended to cluster according to the steroid family. It can thus be observed in Fig. 4 that the androgens (C19 structures) in pink are grouped, the corticosteroids (C21 structures) are also grouped in the blue cluster, and the estrogens (C18 structures) are grouped in the green cluster. Some steroid esters are also highlighted in purple nodes, some of which are not linked to each other because they are grouped with the classification of the steroids, *e.g*. androgen steroids or estrogen steroids. At the bottom, there are also several single nodes because of their unique adduct. Most of the steroids are detected in positive mode, especially in [M + H]^+^, and several steroids can be detected in negative mode, for example, estradiol, 17-glucuronide [M–H]^–^, estrone, 3-sulfate [2 M + Na–2H]^–^. Figure 5 shows the comparison between all the steroids detected in various adducts, including androgen, corticosteroid, estrogen and steroid ester. Most of the steroids were detected in adducts with [M + H]^+^, [M–2H_2_O + H]^+^ and [M–H_2_O + H]^+^. Illustrative chemical representatives of the discussed clusters are displayed in Fig. 6. As an example, Fig. 6A shows the androgens cluster, the structures and the detected adduct of *epi*-19-nortestosterone, 19-nortestosterone, 19-norandrostendione, *epi*-testosterone sulfate and *epi*-testosterone glucuronide are highlighted. These compounds are grouped in one cluster because their fragmentation leads to similar patterns. An interesting example is that of *epi*-testosterone glucuronide, and it could be detected in four adducts, [M + H]^+^, [M–C_6_H_8_O_6_ + H]^+^, [M–C_6_H_8_O_6_–H_2_O + H]^+^ and [M + Na]^+^. As expected, the molecular network cluster of 19-nortestosterone allowed the dereplication of previously known metabolites, including 19-noretiocholanolone, *epi*-testosterone, 19-norandrostendione and 19-norandrosterone. For illustration purposes, another two examples are in Fig. 6B and 6C, estradiol 3-glucuronide, 17-sulfate and estradiol 17-glucuronide could only be detected in positive mode and linked with estradiol 17-glucuronide in the cluster. Dexamethasone is also an interesting compound, which can also be detected in different adducts and form into a cluster with other corticosteroids, such as cortisone, prednisolone, and prednisone. Molecular families are clusters of molecules whose structures are expected to be similar to each other, which gives multiple advantages to further metabolomics data analysis.

### Molecular network construction based GC-HRMS database

**Fig. 7:**
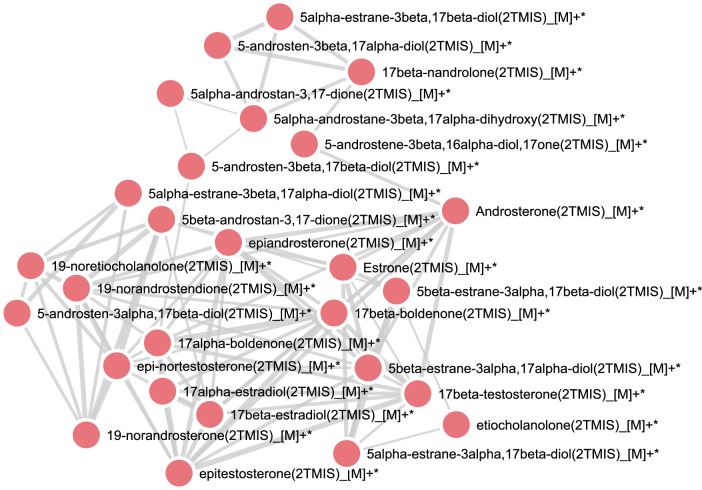
Molecular network of steroid reference compounds generated by GNPS-GC. The cosine score is 0.5.

The database of GC-HRMS contains the formulae, retention time, SMILES, MS spectra and the molecule mass of steroids with two TMS of 27 reference steroids. These steroids could be separated from 13.2 to 15.16 min on GC-Q-Exactive. The majority of GC-MS platforms used in metabolomic studies operate with EI ionisation, and the identification process involves, in addition to the retention time, the specificity associated with the fragmentation process, which depends on the molecular structure^45^. Early MN strategies were developed focusing on LC-ESI-MS data, which differ significantly from traditional GC-EI-MS data. Recently, algorithmic auto-deconvolution of GC-MS data has enabled molecular networking with GNPS. In the GC-MS molecular networking based on GNPS, an essential step for GC-MS data processing, the multiple signals from these highly fragmented spectra must be deconvoluted into individual analyte EI-MS^27,46^. In analysing all GC-MS data, spectral deconvolution is the process of separating fragmentation ion patterns for each eluting molecule into a composite mass spectrum. Annotation of GC-MS data is achieved by matching the deconvoluted fragmentation spectra against reference spectral libraries of known molecules. Currently, GC-EI-MS has significantly higher resolution in chromatography and higher reproducibility in ionization, which allows good prospects for applying GC-GNPS in non-targeted approaches. Therefore, the GC-GNPS approach was used to study a set of steroids in the present study. Figure 7 depicts the molecular network generated by GC-GNPS, involving 27 steroid reference compounds characterized on GC-Q-Exactive, which was visualized in Cytoscape. Each node in the molecular network represents a unique mass spectrometry feature obtained by spectral deconvolution. Since the precursor ion signal is absent on the mass spectrum obtained in GC-HRMS due to energetic ionization by EI and subsequent extensive fragmentation, a list of spectral matches is more likely to contain erroneous annotations (isomers or related isobars) when searching the fragmentation spectra against the GNPS GC libraries. Consequently, the annotations presented in this work were based on GNPS reference libraries and specific libraries (molecular ions, retention time and chemical ionization with MS spectra) generated as part of this study.

Because the molecular ion is absent in GC-EI data, the molecular networks are created through the spectral similarity of the deconvolved fragmentation spectrum without considering the molecular ion in the GNPS GC-MS pipeline workflow. The clustering patterns of the cosine similarity networks in the GC-EI database are mainly determined by structural similarity. In the molecular network of Fig 0.7, nandrolone and its known metabolites, *e.g*. 19-norandrosterone, 19-noretiocholanolone, 19-nroandrostendione and five estranediols (*e.g*. 5β-estrane-3α, 17β-diol; 5β-estrane-3α, 17α-diol) are clustered together. Since steroids comprise a diverse family with similar structures, and androgen steroids share common sub-structures, this explains why they are connected and clustered together. So, this molecular network further guides the annotation at the structural similarity and family level by utilizing information from connected nodes. The proposed molecular network and database provide new insight into investigating steroid metabolomics on GC-HRMS.

### LC-HRMS and GC-HRMS Molecular Networks fusion

The fusion of the LC-MS/MS and GC-MS molecular networks was performed manually based on standard compound consistency. Formerly, nodes related to the same compound, analyzed using both methods were manually connected.

### LC-HRMS and GC-HRMS data sharing and reuse

**Fig. 8:**
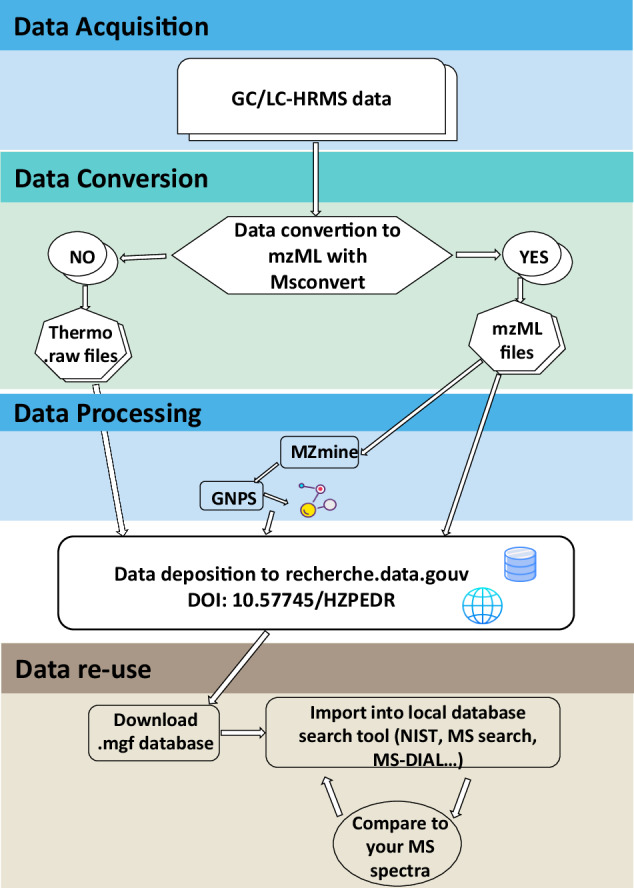
The workflow of data conversion and deposition.

The generated HRMS and extracted MS/MS spectra in the present study have been made public through 10.57745/HZPEDR. All files are public and be opened with dedicated software (Thermo Xcalibur for.raw files and many others for mzML files (mzMine, MS-DIAL, R…). Spectral databases can be reused easily in identification software, such as MSsearch, EntropySearch or MZmine. The workflow used for data conversion and deposition is shown in Fig. 8.

## Data Records

The data set is available at [recherche.data.gouv.fr]^47^, including raw data characterized on both LC-Q-Exactive and GC-Q-Exactive, mzML files of associated raw data, two batches of steroid information, and two steroids databases. It can be accessed through the website link: https://entrepot.recherche.data.gouv.fr/dataset.xhtml?persistentId=doi:10.57745/HZPEDR or (10.57745/HZPEDR).

### Metadata

The two databases of steroid standards acquired on both LC-Q-Exactive and GC-Q-Exactive are recorded with a variety of details, including instrument details, chemical formula, adduct, polarity, retention time, accurate mass, MS1 and MS2 spectra, SMILES, etc. The metadata are available on recherche.data.gouv.fr, with 10.57745/HZPEDR.

## Technical Validation

### Validation of database compounds

**Fig. 9:**
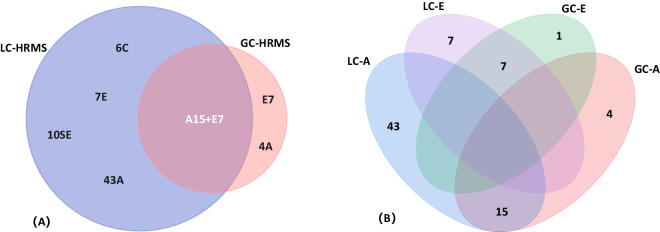
Characterization of all steroid reference compounds using LC-HRMS and GC-HRMS. A: Androgens, E: Estrogens; C: Corticosteroids; SE: Steroid esters; 88 steroid standards were characterized on LC-HRMS, and 27 steroids were characterized on GC-HRMS. Figure (**A**) shows the distribution of standards injected on LC and GC, A and E were characterized on both instruments, while C and SE were injected on LC-HRMS. Figure (**B**) shows the intersection of A and E injected on both two instruments.

Some of the most commonly used preparations in production animals include testosterone propionate in combination with estradiol benzoate or compounds such as 19-nortestosterone (17β-nandrolone) and boldenone. It is for these reasons that in the present study, these steroids were selected to carry out the proof of concept to evaluate the interest of molecular networks in characterizing their metabolisms. In order to consider the broadest possible steroid universe around these target compounds, we chose to construct a network with 88 steroid standards, including androgens, estrogens and corticosteroids, characterized by LC-HRMS. Additionally, 27 steroid standards were characterized on GC-HRMS. Figure 9 shows the number and relationship of steroid standards characterized by the two instruments. For example, 17β-nandrolone and its known metabolites in bovine urine, *e.g. epi*-nandrolone, 19-noretiocholanolone, norandrostenedione and estranediols, were both selected for study on both analytical platforms, depending on their physicochemical properties that make them amenable to both techniques. The analyses of steroid standards were carried out based on optimized analytical methods available in the laboratory. Structures, chemical formula, SMILES, and accurate mass were retrieved from the literature and Pubchem. The obtained mass spectra, exact mass, and retention time were inspected to verify the annotations of the molecular networks.

### Network fusion

**Fig. 10:**
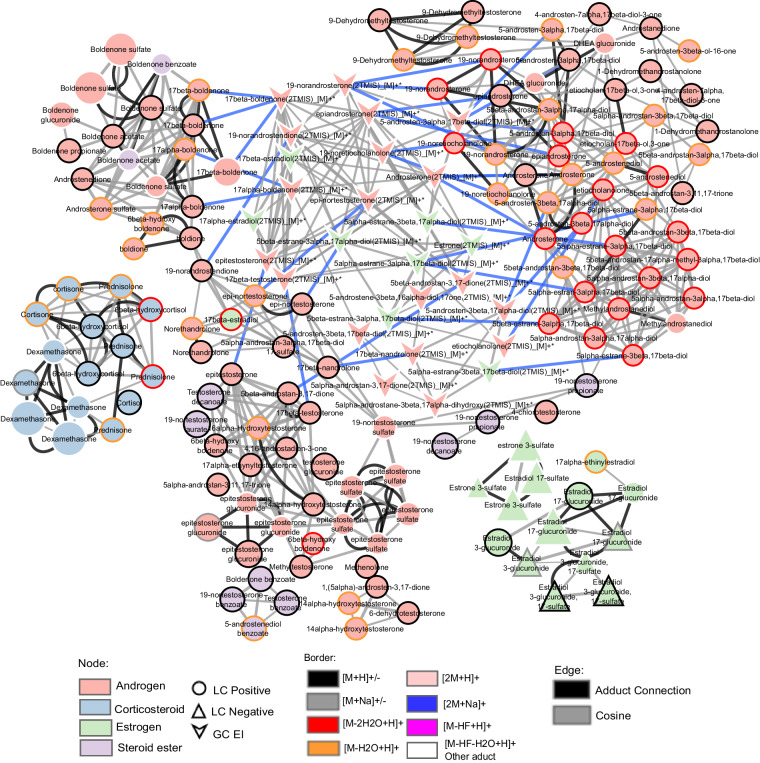
Network merged from both GC-HRMS and LC-HRMS networks. The cosine scores of LC-HRMS and GC-HRMS are 0.5, and the cosine score between the two molecular networks is 1. The “V” nodes represent steroids characterized by GC-HRMS, the ellipse nodes represent steroids analysed by LC Positive, and triangle nodes represent steroids analysed by LC Negative. The blue line represents the connection between the same steroid characterized by LC-HRMS and GC-HRMS.

The network fusion approach refers to the integration of multiple types of metabolomics data, such as liquid chromatography and gas chromatograph mass spectrometry data or other omics data into a unified network. Network fusion in metabolomics offers a powerful approach to integrate and analyse diverse metabolomics data sources, which provides insights into the metabolic pathways and biomarkers findings^48^. Consequently, the integration of both networks is particularly innovative, providing new insights into the metabolomics of steroids, in addition to reporting possible complementarity between both ionisations in a MN strategy perspective. Thus, after the generation of molecular networks using data from both instruments (using GNPS), based on the large dataset containing more than 120 steroid standards characterized on both LC-HRMS and GC-HRMS (Fig. 9). To go further in creating a more global annotation tool relevant for steroidome studies, the two networks were fused. The fusion of the LC-MS/MS and GC-MS molecular networks was performed manually based on standard compound consistency. Formerly, nodes related to the same compound, analysed using both methods were manually connected. The fused networks (Fig. 10) were based on the 22 steroid compounds, *e.g*., androgens and estrogens, characterized on both platforms, and the blue edges represent the connection between the LC and GC networks. This network fusion, as proposed in Fig. 10, highlights the complementarity between both data sources. As an example, 17β-boldenone and 19-norandrosterone, both androgens, are not connected through the LC-HRMS/MS network. In comparison, they are connected within the GC-MS network. Such complementarity is clearly in Fig. 10, where the GC-MS (triangle) nodes are groups and located in the middle of the global network in between two sub-clusters of the main androgen cluster. Due to estrogens *e.g*. estradiol and estrane-diols are the metabolites of 17β-nandrolone (androgen)^14,33^, it is possible for estrogen compounds to share similar fragmentation patterns or mass spectral features with certain androgens, they may indeed be connected within the same molecular network. This result highlights the expected differences between the fragmentation mechanisms according to ESI/EI MS. Thus, grouping data from both origins may add additional connectivity and thus interpretability.

Data integration is a current challenge that aims to bridge the gap between multi-omics or different platforms in metabolomics, which generate vast amounts of data. The objective is to improve global understanding of biological systems by aggregating different data sets. Although the integration of data makes it possible to explore the scientific question, combining these types of data represents a huge challenge. In particular, each platform has its own characteristics, which complicates the integration process. Difficulties such as the diversity in the size of datasets and their format, as well as the lack of standardisation in data communication and storage, make it difficult to combine datasets from different suppliers and instruments. In addition, missing data, noise in different types of data, and the correspondence and correlation between measurements from different platforms can also create difficulties. In fact, not only standardised protocols and advanced data processing algorithms, but also new tools and software are emerging to address these challenges and difficulties, facilitating the integration of data from various platforms or sources. In this study, merging the networks was facilitated by the consistency of the steroid compounds characterised by the two instruments, which also came from the same supplier, so that the data was in a similar format.. The fused molecular network was constructed using an edge.

### Database application to urine

Technical validation was also achieved by applying the database and molecular network to annotate steroids in a biological environment, bovine urine, a natural excretion matrix rich in steroids, was analysed by GC-HRMS. Raw data from urine samples were converted to.mzML files with MSconvert software. FTP client (WinSCP for Windows) was used to upload.mzML files to MassIVE. A repository scale analysis infrastructure for GC-MS data enables the creation of networks within the GNPS molecular networking interface, and the community infrastructure can be accessed at https://gnps.ucsd.edu. The GC-MS Hub platform was used for data processing deconvolution, and the.mgf file, including the deconvoluted spectra, was generated by GNPS. Then, the spectra list was sent to the library search to search against the GNPS public libraries and personal libraries generated in this study. The MSHUB-GC job and library search job are available on the GNPS platform, MSHUB-GC job: https://gnps.ucsd.edu/ProteoSAFe/status.jsp?task=7737ac63afc74820b57fcc480cf26639; Library search job: https://gnps.ucsd.edu/ProteoSAFe/status.jsp?task=6d0b0f1772a04da5847d0ee9a0272df7. A molecular network of urine samples was visualized in Cytoscape software.

**Fig. 11:**
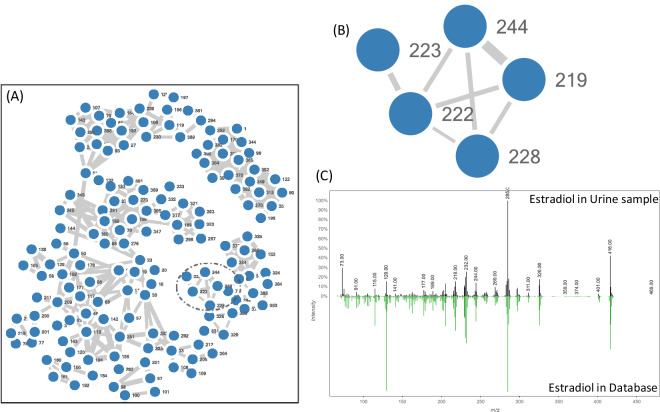
Molecular network of urine sample characterized on GC-HRMS and estradiol mass spectra comparison.

MN methodology was then applied to the data acquired, as shown in Fig. 11. Figure 11A illustrates the overall molecular network of urine samples, while Fig. 11B is a zooming-in corresponding to the subnetwork of a given cluster, and Fig. 11C is the comparison of estradiol annotation spectra between the library and in the urine sample. This urine molecular network was generated by GC-GNPS library search after MSHUB deconvolution. An annotation of estradiol is highlighted after a search of the urine spectra with the library. The cosine similarity of estradiol between the library and urine sample is 0.9. This result shows the potential of the approach as a new tool for annotating steroid compounds in complex matrices. It also meets expectations in terms of identification in non-targeted steroidomics studies.

Due to the complexity of steroid metabolism in urine, many unknown metabolites could be formed. Non-targeted strategies based on LC-HRMS or GC-HRMS provide a new research perspective and opportunities for deeper investigation of steroid metabolomics and finding new biomarkers or interests. In the present study, two steroid standard databases were generated: 88 reference steroids characterized by LC-HRMS and 27 reference steroids characterized by GC-HRMS. The databases include retention time, formulas, adducts, etc. Then, GNPS served as a data analysis infrastructure with MN and was used to construct molecular networks of steroids in two databases respectively. The global steroid network of LC-HRMS data involves 198 detected adducts of steroids and steroids from the same family clustered together, *e.g*., androgens, estrogens, and corticosteroids. And the clusters of steroids from different families are separated. In the molecular network of GC-HRMS data, androgens and estrogens also formed two clusters. To our present knowledge, this is the first research using GNPS-MN to study steroid metabolomics. In addition, few urine samples were characterized on GC-HRMS, and the database generated in the current study were applied to investigate steroids in urine samples. An annotation of estradiol is highlighted, thanks to the database and technologies of GNPS, we have the capability to detect and annotate steroids in bovine urine samples. Therefore, based on the results of available studies, MN could be considered as a proof of concept of a new strategy to evidence steroid chemical structure similarities and could be used to investigate new steroid biomarkers of interest. Moreover, the two libraries of steroids are also regularly extended by the scientific community of new reference spectra, which could strengthen MS annotations.

## Data Availability

Each spectra in.mgf format were merged with an R script using the R 3.6.0 language, and it is publicly available on GitHub, https://github.com/Ting1217/Feature-Merge-R.
